# Supplementary material for: Computational joint action: Dynamical models to understand the development of joint coordination
Source: PLoS Comput Biol. 2024 Oct 22;20(10):e1011948. doi: 10.1371/journal.pcbi.1011948 (PMC11530078; doi:10.1371/journal.pcbi.1011948)
Supplement: S1 File — (PDF) [file pcbi.1011948.s001.pdf]

Supplementary Note

Model definition and identification procedure

[1]Cecilia De Vicariis, [1,2]Vinil T. Chackochan, [1]Laura Bandini,
[1]Eleonora Ravaschio, and [1]Vittorio Sanguineti

[1]*Department of Informatics, Bioengineering, Robotics and Systems Engineering*
University of Genoa, Genoa (ITALY)
[2]*Odstock Medical Ltd, Salisbury (UK)*

This document contains the analytical derivation of three inter-related aspects of the joint action model: (i) definition of the model and derivation of the action selection and strategy choice procedure; and (ii) identification of model parameters from joint action time series.

Model definition

The aim of this section is to derive the defining equations for action selection and strategy choice as defined in the main paper.

The optimal action of the i-th player at time t, $u_i(t)$ minimizes the expected cost $\bar{J}_i(u_i) = E\{J_i(u_i, u_{-i})\}$, where the average is calculated over the partner's action, u_{-i}:

$$\bar{J}_i(u_i) = E\{J_i(u_i, u_{-i})\} = \int J_i(u_i, u_{-i}) \cdot p(u_{-i}) \cdot du_{-i} \tag{1}$$

When multiple equilibria exist, each player needs to select strategy s_i and action u_i based on the task and on their belief in partner action. This is achieved by minimizing the expected cost $\bar{J}_i(u_i, s_i) = E\{J_i(u_i, u_{-i}, s_i)\}$. However, the i-th player only knows their partner's action through the history of sensory observations $D_i(t) = \{y_i(1) \dots y_i(t)\}$, so that the average is made over $p(u_{-i}|D_i(t))$. The quantity $p(u_{-i}|D_i(t))$ results from the Kalman integration algorithm.

Let $\hat{u}^-_{-i}$ and $\hat{u}^+_{-i}$ be the estimates of partner action, respectively before and after the sensory information is available, i.e. after the action has taken place (hereafter, 'prior' and 'posterior' estimates). The posterior estimate combines the prior estimate and the sensory information:

$$\begin{aligned} &\hat{u}^+_{-i}(t) = \hat{u}^-_{-i}(t) + K_i(t)\left[y_i(t) - H^i \cdot \hat{u}^-_{-i}(t) - L^i \cdot u_i(t)\right] \\ &P^+_i(t) = \left[I - K_i(t)H^i\right] P^-_i(t) \end{aligned} \tag{2}$$

where the Kalman gain is defined as:

$$K_i(t) = P^-_i(t) H^{iT} \left[H^i P^-_i(t) H^{iT} + \Sigma^i_y\right]^{-1} \tag{3}$$

is calculated iteratively in terms of the covariance of the prior estimate. The prior estimate (mean and covariance) propagates to the next trial by accounting for the player's retention rate:

$$\begin{aligned} &\hat{u}^-_{-i}(t+1) = A^i \cdot \hat{u}^+_{-i}(t) \\ &P^-_i(t+1) = A^i P^+_i(t) A^{iT} + \Sigma^i_x \end{aligned} \tag{4}$$

Hereafter, we will refer to the prior estimate of partner action as the 'partner model': $x_i(t) \equiv \hat{u}^-_{-i}(t)$ and $P_i(t) \equiv P^-_i(t)$. Equations 2 and 4 can be merged into:

$$\begin{aligned} x_i(t+1) &= A^i \left\{ x_i(t) + K_i(t) \cdot \left[y_i(t) - H^i \cdot x_i(t) - L^i \cdot u_i(t) \right] \right\} \\ P_i(t+1) &= A^i \left[I - K_i(t) H^i \right] P_i(t) {A^i}^T + \Sigma^i_x \end{aligned} \tag{5}$$

Equations 3 and 5 completely specify the temporal evolution of the partner model as a function of sensory information and own actions.

At each trial the partner model provides the expected value $x_i(t)$ of the partner's action and its covariance matrix $P_i(t)$, so that the expected cost can be expressed in terms of these quantities:

$$\begin{aligned} \bar{J}_i(u_i, s_i, x_i) &= \tfrac{1}{2} u_i^T \cdot R^i_{ii}(s_i) \cdot u_i + \left[x_i^T \cdot R^i_{i-i}(s_i) + r^i_i(s_i) \right] \cdot u_i + \\ & \quad r^i_{-i}(s_i) \cdot x_i + z^i(s_i) + \tfrac{1}{2} \mathrm{tr} \left[R^i_{-i-i}(s_i) \cdot P_i \right] + \tfrac{1}{2} x_i^T \cdot R^i_{-i-i}(s_i) \cdot x_i \\ &= \tfrac{1}{2} \left[u_i - u^*_i(s_i, x_i) \right]^T \cdot R^i_{ii}(s_i) \cdot \left[u_i - u^*_i(s_i, x_i) \right] + q(s_i, x_i) \end{aligned} \tag{6}$$

where

$$u^*_i(s_i, x_i) = -R^i_{ii}(s_i)^{-1} \cdot \left[R^i_{i-i}(s_i) \cdot x_i + r^i_i(s_i) \right] = H_c(s_i) \cdot x_i + h_c(s_i) \tag{7}$$

and

$$\begin{aligned} q(s_i, x_i, P_i) = & -\tfrac{1}{2} u^*_i(s_i, x_i)^T \cdot R^i_{ii}(s_i) \cdot u^*_i(s_i) + r^i_{-i}(s_i) \cdot x_i + z^i(s_i) + \\ & \tfrac{1}{2} \mathrm{tr} \left[R^i_{-i-i}(s_i) \cdot P_i \right] + \tfrac{1}{2} x_i^T \cdot R^i_{-i-i}(s_i) \cdot x_i \end{aligned} \tag{8}$$

Therefore, optimal action u_i and strategy s_i are determined by minimizing the expected cost with respect to both the action u_i and the strategy s_i:

$$(u^*_i, s^*_i) = \arg \min_{(u_i, s_i)} \bar{J}_i(u_i, s_i, x_i) \tag{9}$$

To account for variability in action selection – i.e. players not behaving in a deterministic way, or having an imperfect knowledge of their cost function – we assume that the selection of both strategy and action is in fact a stochastic process. The expected cost can be interpreted as an energy term, so that the joint probability of u_i and s_i given the partner model can be expressed as a Boltzmann probability density function:

$$p(u_i, s_i | x_i) = \frac{1}{Z} e^{-\frac{1}{\lambda^i} \bar{J}_i(u_i, s_i, x_i)} \tag{10}$$

where λ^i reflects action selection 'temperature', with greater λ^i leading to greater randomness.

If the matrices $R^i_{ii}(s_i)$ are positive definite, the normalization factor is calculated as:

$$Z = \sum_{j=1}^{M} \int e^{-\frac{1}{\lambda^i} \bar{J}_i(u_i, S^i_j, x_i)} du_i = (2\pi)^{d/2} \sqrt{\lambda^i} \sum_{j=1}^{M} |R^i_{ii}(S^i_j)|^{-1/2} \cdot e^{-\frac{q(S^i_j, x_i)}{\lambda^i}} \tag{11}$$

By substituting into Eq. 10, we get:

$$p(u_i, s_i = S^i_k | x_i) = p(u_i | s_i = S^i_k, x_i) \cdot \Pr(s_i = S^i_k | x_i) \tag{12}$$

The action probability given the partner model x_i and the strategy $s_i = S^i_k$ has a multivariate Gaussian distribution, with mean $u^*_i(S^i_k, x_i)$ – see Eq. 7 – and covariance $\Sigma^i_u(S^i_k) = \lambda^i \cdot R^i_{ii}(S^i_k)^{-1}$:

$$p(u_i | s_i = S^i_k, x_i) = (2\pi)^{-d/2} |\Sigma^i_u(S^i_k))|^{-1/2} \exp \left\{ -\tfrac{1}{2} \cdot \left[u_i - u^*_i(S^i_k) \right]^T \Sigma^i_u(S^i_k)^{-1} \left[u_i - u^*_i(S^i_k) \right] \right\} \tag{13}$$

whereas the strategy prior, i.e. the probability to select strategy $s_i = S_k^i$ given the partner model x_i, is calculated as:

$$\Pr(s_i = S_k^i | x_i) = \frac{|R_{ii}^i(S_k^i)|^{-1/2} \cdot e^{-\frac{1}{\lambda^i} q(S_k^i, x_i)}}{\sum_{j=1}^{M} |R_{ii}^i(S_j^i)|^{-1/2} \cdot e^{-\frac{1}{\lambda^i} q(S_j^i, x_i)}} \tag{14}$$

In conclusion, with positive definite $R_{ii}^i(s_i)$ the action u_i given the partner model x_i has a Gaussian Mixture distribution:

$$p(u_i|x_i) = \sum_k p(u_i, s_i = S_k^i | x_i) = \sum_k p(u_i | s_i = S_k^i, x_i) \cdot \Pr(s_i = S_k^i | x_i) \tag{15}$$

Model identification

Parameters identification procedure

The model can be used to simulate the evolution of a particular game, or as an analytical tool: given a time series of players' actions, it is possible to estimate the model parameters which best describe each player in a dyad. To do this, we need to derive a fitting procedure that maximizes the model likelihood with respect to the time series of both players' actions, i.e. $\mathcal{D} = \{u_1(t), u_2(t), t = 1, \ldots, T\}$. The procedure has to be carried out separately for each player in the dyad to estimate the two players' respective model parameters. Derivation of the parameter identification procedure requires a view of the model from the experimenter's point of view as opposed to the subject's viewpoint which has been used in the main paper. From the experimenter's point of view both actions are observed, but all other model variables, i.e. the partner model x_i, P_i , the sensory input, y_i, and the strategy choice, s_i, are not directly observable. The experimenter's viewpoint is depicted in Figure 1.

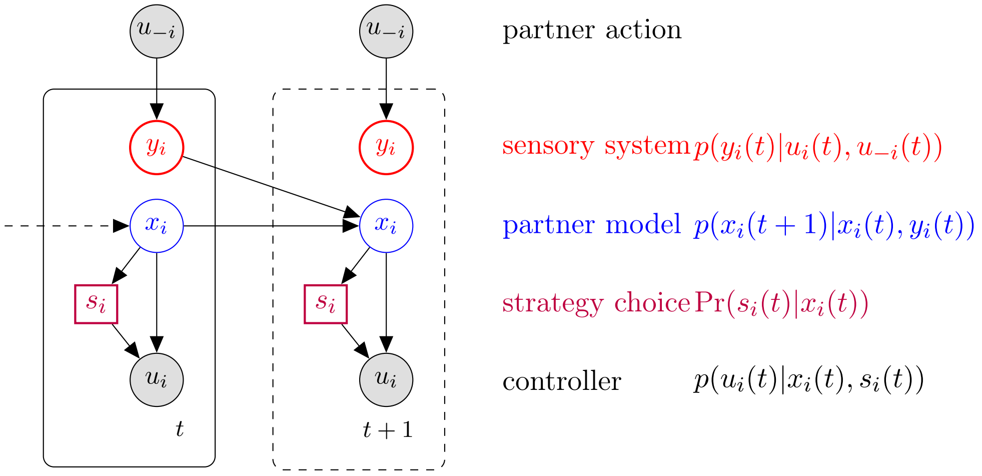

Figure 1: Probabilistic graphical model of a single joint action player from the experimenter's point of view. At each time step, Player i receives information about partner action (u_{-i}) through their sensory system (y_i). This information is used to predict the next partner action (partner model, x_i). The partner model is used to select the actual action (u_i) which also depends on the strategy choice (s_i). The latter decision is also affected by the player's knowledge about the partner. For each player, sensory input (y_i), partner model (x_i) and strategy choice (s_i) are latent variables; actions (u_i and u_{-i}) are observable variables (denoted by shaded nodes)

For parameter identification purposes, the model of the i-th player – see Eq.5 and Eq.15 – can be reformulated as

$$\begin{array}{l} x_i(t+1) = F^i(t) \cdot x_i(t) + G^i(t) \cdot u_{-i}(t) + w_i(t) \\ P_i(t+1) = F^i(t) P_i(t) A^{iT} + \Sigma_x^i \\ p(u_i|x_i) = \sum_k p(u_i, s_i = S_k^i | x_i) = \sum_k p(u_i | s_i = S_k^i, x_i) \cdot \Pr(s_i = S_k^i | x_i) \end{array} \tag{16}$$

where the true partner action $u_{-i}(t)$ is the model's external input, $x_i(t)$ is the internal state (partner model) and $u_i(t)$ is the output; the noise term $w_i(t) \sim N(0, \Sigma_w^i)$ can be interpreted as a process noise; and:

$$\begin{array}{l} K_i(t) = P_i(t) \cdot H^{iT} \left[H^i P_i(t) H^{iT} + \Sigma_y^i \right]^{-1} \\ F^i(t) = A^i \left[I - K_i(t) \cdot H^i \right] \\ G^i(t) = A^i \cdot K_i(t) \cdot H^i \\ \Sigma_w^i(t) = A^i \cdot K_i(t) \cdot \Sigma_y^i \cdot K_i(t)^T \cdot A^{iT} \end{array} \tag{17}$$

The model parameters $b^i = \left\{ \mu^i, P_0^i, A^i, \Sigma_x^i, \Sigma_y^i, \lambda_1^i, a^i \right\}$ can be identified using a non-linear optimization method. To do so, we reformulate the model in predictor form [1]. We first consider the special case of a task described by a single quadratic cost, for which model output is simply $u_i(t) = H_c^i \cdot x_i + h_c^i + v_i(t)$, with $v_i(t) \sim N(0, \Sigma_v^i(t))$ and $\Sigma_v^i(t) = \lambda^i(t) \cdot {R_{ii}^i}^{-1}$ – see Eq.13. The posterior estimate of $x_i(t)$ given the data until time t, $\mathcal{D}_t$, is expressed as:

$$\begin{array}{lll} \hat{x}_i^+(t) & = E\{x_i(t)|\mathcal{D}_t\} & = \hat{x}_i^-(t) + W^i(t) \left[u_i(t) - H_c^i \cdot \hat{x}_i^-(t) - h_c^i \right] \\ \hat{P}_i^+(t) & = \mathrm{cov}\{x_i(t)|\mathcal{D}_t\} & = \left[I - W^i(t) \cdot H_c^i \right] \cdot \hat{P}_i^-(t) \end{array} \tag{18}$$

where:

$$W^i(t) = \hat{P}_i^-(t) \cdot H_c^{iT} \cdot \left[H_c^i \cdot \hat{P}_i^-(t) \cdot H_c^{iT} + \Sigma_v^i(t) \right]^{-1} \tag{19}$$

The update equations for the corresponding prior estimates are:

$$\begin{array}{lll} \hat{x}_i^-(t+1) & = & F^i(t) \cdot \hat{x}_i^+(t) + G^i(t) \cdot u_{-i}(t) \\ \hat{P}_i^-(t+1) & = & F^i(t) \cdot \hat{P}^+(t) \cdot F^i(t)^T + \Sigma_w^i \end{array} \tag{20}$$

For a given b^i, we use Eqs. 18-20 to estimate the sequence $\left\{ \hat{x}_i^-(t), \hat{P}_i^-(t) | t = 1, \ldots, T \right\}$. The predicted action $u_i(t)$ given D_{t-1} has the following mean and covariance:

$$\begin{array}{lll} \hat{u}_i^-(t) = E\{u_i(t)|\mathcal{D}_{t-1}\} & = & H_c^i \cdot \hat{x}_i^-(t) + h_c^i \\ Q_i^-(t) = \mathrm{cov}\{u_i(t)|\mathcal{D}_{t-1}\} & = & H_c^i \cdot \hat{P}_i^-(t) \cdot H_c^{iT} + \Sigma_v^i(t) \end{array} \tag{21}$$

so that the expected log-likelihood is calculated as:

$$\mathcal{L}(b^i) = -\frac{1}{2} \sum_{t=1}^{T} \left\{ \log \left| Q_i^-(t) \right| + \left[u_i(t) - \hat{u}_i^-(t) \right]^T \cdot Q_i^-(t)^{-1} \cdot \left[u_i(t) - \hat{u}_i^-(t) \right] \right\} \tag{22}$$

This function can be maximized numerically [1].

In the case of multiple quadratic costs, model output u_i has a Gaussian Mixture distribution – see Eq. 16. In this case, the model can be seen as a linear system with a switching 'measurement' model. Eq.18 becomes [2]:

$$\begin{array}{lll} \hat{x}_i^+(t) & = E\{x_i(t)|D_t\} & = \hat{x}_i^-(t) + \sum_k \pi_i^k(t) \cdot W_k^i(t) \left[u_i(t) - H_c^i(S_i^k) \cdot \hat{x}_i^-(t) - h_c^i(S_i^k) \right] \\ \hat{P}_i^+(t) & = \mathrm{cov}\{x_i(t)|D_t\} & = \sum_k \pi_i^k(t) \cdot \left[I - W_k^i(t) \cdot H_c^i(S_i^k) \right] \cdot \hat{P}_i^-(t) \end{array} \tag{23}$$

where

$$\pi_i^k(t) = \Pr(s_i = S_k^i | u_i, x_i) = \frac{p(u_i | s_i = S_k^i, x_i) \cdot \Pr(s_i = S_k^i | x_i)}{\sum_j p(u_i | s_i = S_j^i, x_i) \cdot \Pr(s_i = S_j^i | x_i)} \tag{24}$$

is the posterior probability of the strategy $s_i(t) = S_k^i$, given the partner model $x_i(t)$ and the observation $u_i(t)$. Quantities $p(u_i|s_i = S_j^i, x_i)$ and $\Pr(s_i = S_j^i|x_i)$ are calculated from Eqs. 13-14, and

$$W_k^i(t) = \hat{P}_i^-(t) \cdot H_c^i(S_i^k) \cdot \left[H_c^i(S_i^k) \cdot \hat{P}_i^-(t) \cdot H_c^i(S_i^k)^T + \Sigma_v^i(S_i^k) \right]^{-1} \tag{25}$$

In this case, the expected log-likelihood becomes:

$$\mathcal{L}(b^i) = \sum_{t=1}^{T} \log \left\{ \sum_k \pi_i^k(t) \cdot \left| Q_i^-(S_i^k) \right|^{-1/2} \cdot e^{-\frac{1}{2}\left[u_i(t) - \hat{u}_i^-(S_i^k)\right]^T \cdot Q_i^-(S_i^k)^{-1} \cdot \left[u_i(t) - \hat{u}_i^-(S_i^k)\right]} \right\} \tag{26}$$

with:

$$\begin{array}{lcl} \hat{u}_i^-(S_i^k) = E\{u_i(t) | s_i = S_k^i, \mathcal{D}_{t-1}\} & = & H_c^i(S_i^i) \cdot \hat{x}_i^-(t) + h_c^i(S_k^i) \\ Q_i^-(S_i^k) = \mathrm{cov}\{u_i(t) | s_i = S_k^i, \mathcal{D}_{t-1}\} & = & H_c^i(S_k^i) \cdot \hat{P}_i^-(t) \cdot H_c^i(S_k^i)^T + \Sigma_v^i(S_k^i) \end{array} \tag{27}$$

Performance and limitations

We first tested whether the identification procedure worked correctly. We used the toy example (sensorimotor version of the Stag Hunt game, see the main text) to simulate a joint action time sequence for both players in a dyad. We then used these same sequences to estimate the model parameters. We initialized the fitting procedure using random parameter values. Successfully recovering the true parameter values in a player model requires that the model input – time series of partner actions – exhibits sufficient variability to excite the model's dynamic modes of response (persistent excitation condition). To ensure this, for each player in a dyad we first simulated a player's action time series in response to a partner's sequence of random actions. We found that under these conditions the fitting procedure is indeed capable of recovering the true values of the parameters – see Table 1. We then looked at simulated joint action time sequences, in which each player's action time sequence was used as output and the corresponding sequence of partner actions was taken as input. In this case, the model parameters could only be correctly estimated in the simulation where $\Sigma_x = 0.1$ and $\Sigma_y = 10$ – the only one that exhibited some temporal variability in both players' actions. This finding was not unexpected, and it can be assumed that action time sequences from real joint action trials satisfy the persistent excitation condition. In this condition, we found an average $R^2 = 0.9716$.

Does the model fit the data well?

To verify that the resulting model fits the data well, we compared the actual data – the available action time series of both players in each dyad, i.e. $u_1(t)$ and $u_2(t)$, from the 2-VP experiments – with the predictions of the fitted model. Specifically, the actual data were compared with: (i) the model predictions generated within the fitting procedure – the $\hat{u}_i^-(t)$ in Eqns. 22 and 26; (ii) the action time series obtained by simulating the model of a single player, by keeping fixed the partner's action time series; and (iii) the action time series obtained by simulating the whole dyad, so that at each trial the model generates both players' actions. While the predictions (i) represent the optimal fit of the model to data, quantities (ii) and (iii) reflect the stochastic nature of the model. We used the coefficient of determination, R^2 to quantify the mismatch between the actual action at time t, $u_i(t)$, and the corresponding model prediction.

Parameters	Average Estimated Values	True Values	Absolute Error
A	0.97799	0.99	0.012014
Σ_x	0.11177	0.1	0.011771
Σ_y	10.438	10	0.43778
x_0	-6.1099e-14	0	6.1099e-14
$\hat{P}_0$	5.007e-11	4.4409e-16	5.007e-11
λ	0.10368	0.1	0.0036806
a_λ	0.999	0.999	3.7217e-06

Table 1: Parameters estimated for the 8 simulated dyads, true parameters values used for the simulations and absolute error

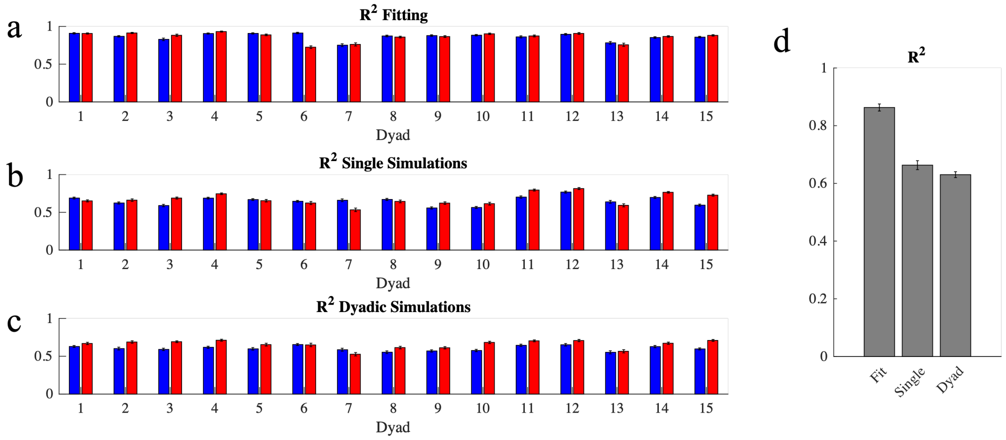

Figure 2: Fitting performances. Average and standard errors of the R^2 are reported for each player within each dyad. Blue and red bars refer respectively to Player 1 and Player 2. a) R-squared is reported for the estimated trajectories in the fitting procedure. b) R-squared is reported for the actions obtained by simulating the single players. c) R-squared is reported for the actions obtained by simulating the whole dyads. d) Average and standard error is reported at the population level for the three conditions.

References

[1] Gupta N, Mehra R. Computational aspects of maximum likelihood estimation and reduction in sensitivity function calculations. IEEE Transactions on Automatic Control. 1974;19(6):774–783.

[2] Shumway RH, Stoffer DS. Dynamic Linear Models With Switching. Journal of the American Statistical Association. 1991;86(415):763–769.
